# Variables that influence HIV-1 cerebrospinal fluid viral load in cryptococcal meningitis: a linear regression analysis

**DOI:** 10.1186/1758-2652-12-33

**Published:** 2009-11-11

**Authors:** Diego M Cecchini, Ana M Cañizal, Haroldo Rojas, Alicia Arechavala, Ricardo Negroni, María B Bouzas, Jorge A Benetucci

**Affiliations:** 1 Infectious Diseases Department, Infectious Diseases Hospital "Francisco J Muñiz", Buenos Aires, Argentina; 2 Virology Unit, Infectious Diseases Hospital "Francisco J Muñiz", Buenos Aires, Argentina; 3 Mycology Unit, Infectious Diseases Hospital "Francisco J Muñiz", Buenos Aires, Argentina

## Abstract

**Background:**

The central nervous system is considered a sanctuary site for HIV-1 replication. Variables associated with HIV cerebrospinal fluid (CSF) viral load in the context of opportunistic CNS infections are poorly understood. Our objective was to evaluate the relation between: (1) CSF HIV-1 viral load and CSF cytological and biochemical characteristics (leukocyte count, protein concentration, cryptococcal antigen titer); (2) CSF HIV-1 viral load and HIV-1 plasma viral load; and (3) CSF leukocyte count and the peripheral blood CD4+ T lymphocyte count.

**Methods:**

Our approach was to use a prospective collection and analysis of pre-treatment, paired CSF and plasma samples from antiretroviral-naive HIV-positive patients with cryptococcal meningitis and assisted at the Francisco J Muñiz Hospital, Buenos Aires, Argentina (period: 2004 to 2006). We measured HIV CSF and plasma levels by polymerase chain reaction using the Cobas Amplicor HIV-1 Monitor Test version 1.5 (Roche). Data were processed with *Statistix *7.0 software (linear regression analysis).

**Results:**

Samples from 34 patients were analyzed. CSF leukocyte count showed statistically significant correlation with CSF HIV-1 viral load (*r *= 0.4, 95% CI = 0.13-0.63, *p *= 0.01). No correlation was found with the plasma viral load, CSF protein concentration and cryptococcal antigen titer. A positive correlation was found between peripheral blood CD4+ T lymphocyte count and the CSF leukocyte count (*r *= 0.44, 95% CI = 0.125-0.674, *p *= 0.0123).

**Conclusion:**

Our study suggests that CSF leukocyte count influences CSF HIV-1 viral load in patients with meningitis caused by *Cryptococcus neoformans*.

## Background

Invasion of the central nervous system (CNS) occurs early in the course of HIV-1 infection, but the exact mechanisms of HIV-1 entry to the brain are still under debate [1,2]. Although very high levels of viremia occur during primary HIV-1 infection, the circulating virus is unable to penetrate the CNS at this time due to the highly restricted permeability of the blood-brain barrier.

However, the blood-brain barrier is permeable to immune cells, which has led to the proposal that HIV-1 might be transported to the CNS by infected immune cells (Trojan horse hypothesis) [1-4]. The biochemical characteristics of cerebrospinal fluid (CSF), which surrounds brain tissue, may reflect cellular events in brain parenchyma. Therefore, investigations of HIV-1 have used CSF as a surrogate for brain pathophysiologyc events [5,6].

HIV-1 is found in the CSF of most infected individuals at all stages of the disease, including primary infection and the asymptomatic and symptomatic (i.e., occurrence of CNS opportunistic diseases) phases [2,7,8]. It establishes an active and productive infection, triggering an intrathecal cell-mediated immune response characterized by elevated concentrations of β2-microglobulin and neopterin in the CSF.

HIV-1 infection also induces a humoral immune response in the CNS, as measured by an increased immunoglobulin G index. The highest levels of CSF neopterin are found in infected patients with CNS opportunistic infections or AIDS dementia complex, although asymptomatic carriers may also show moderately increased levels [5,9-11]. Therefore, the virus is present at all stages of the disease, irrespective of the development of neurologic symptoms or opportunistic infections [1].

In patients without opportunistic infections, CSF HIV-1 viral load depends mainly on the plasma viral load and the CSF leukocyte count [7,12]. However, little is known about what factors may influence CSF HIV-1 viral load in patients with such infections. For example, no correlation has been found between the viral load in plasma and that in the CSF, although some studies have suggested that cell-free CSF viral load correlates with the number of CSF white cells [8,13].

However, these studies included a low number of patients with different CNS opportunistic infections (e.g., cerebral toxoplasmosis, cryptococcal meningitis, *Cytomegalovirus *encephalitis, progressive multifocal leukoencephalopathy, and tuberculous meningitis) that were analyzed together [13-15]. That is, to the best of our knowledge, no study has considered CSF viral load in the context of a single opportunistic infection.

A more disease-focused approach would avoid such a heterogeneous analysis regarding opportunistic agents, and therefore may better elucidate some of the factors that affect CSF HIV-1 viral load in these diseases. This is particularly important considering that each microorganism has its own virulence factors and a particular pathophysiology that generates an intrathecal immune response that, in turn, may promote CSF HIV-1 replication.

*Cryptococcus neoformans *is a yeast fungus with two unique characteristics: it produces a polysaccharide capsule, and is neurotropic, being one of the most common causes of meningitis in HIV-1 infected patients [16]. The main virulence factor of this pathogen is the capsular polysaccharide antigen (CCPA), which inhibits both the migration of leukocytes from the bloodstream to an inflammatory site (usually the CNS) and the phagocytosis [17,18]. Therefore, in cryptococcal meningitis, CSF pleocytosis may be absent or reduced despite the active CNS infection [19]. Considering these particular characteristics, the variables that influence CSF HIV-1 viral load in this disease, with a focus on the CSF leukocyte count, merit further investigation.

CSF leukocyte count is positively correlated with peripheral blood CD4+ T lymphocyte count in asymptomatic HIV-1 infected patients (i.e., those without CNS opportunistic diseases), but this appears not to be the case in symptomatic patients, possibly due to the low CD4+ T cell counts found in this latter population [4,14]. However, the influence of CD4+ T lymphocyte levels on the development of a cellular inflammatory CSF response has never been investigated in a cohort of patients with cryptococcal meningitis.

*In vitro *studies have demonstrated that a clinically relevant concentration of CCPA enhances HIV-1 production in H9 cells and peripheral mononuclear cells [20]. Considering this relevant *in vitro *interaction, it is necessary to investigate the potential correlation between this microorganism burden (CCPA titers) and CSF HIV-1 viral load in a clinical setting.

In this context, we designed an observational prospective investigation to describe the factors that may influence CSF HIV-1 viral load in patients with meningitis caused by *Cryptococcus neoformans*. The objectives were to evaluate the relationships between: CSF HIV-1 viral load and CSF cytological and biochemical characteristics (e.g., leukocyte count, protein concentration and CCPA titer); CSF HIV-1 viral load and HIV-1 plasma viral load; and CSF leukocyte count and peripheral blood CD4+ T lymphocyte count.

## Methods

We conducted a prospective single-centre observational non-comparative study. We prospectively collected and analyzed pre-treatment, paired CSF and blood samples from 34 antiretroviral-naive HIV-1 positive patients with culture-confirmed cryptococcal meningitis at the Francisco J Muñiz Infectious Diseases Hospital in Buenos Aires, Argentina (period: 2004 to 2006). All procedures were in accordance with this institution's ethical standards and with the Helsinki Declaration of 1975, as revised in 1983.

CSF cytological and biochemical characteristics, including CCPA titer, were analyzed. All samples were routinely cultured on Saboureaud agar plates to detect fungal pathogens, and in other media to detect mycobacteria (Lowenstein-Jensen) and common aerobic bacteria (blood agar). Patients with positive cultures for pathogens other than *Cryptococcus neoformans *or evidence of another simultaneous CNS infection were excluded.

For each patient, we measured CSF HIV-1 and plasma viral loads in the same assay by polymerase chain reaction using the Cobas Amplicor Monitor Test version 1.5 (Roche Diagnostic Systems, Inc, Branchburg, NJ) following the manufacturer's instructions. CCPA titer was measured by latex agglutination (Latex-*Cryptococcus *Antigen Detection System, Immuno-Mycologics, Norman, OK) following the manufacturer's instructions. Peripheral blood CD4+ T lymphocyte count was determined by flow cytometry (Cytoron Absolute, Ortho Diagnostic Systems, Johnson & Johnson Co, Raritan, NJ).

Linear regression analysis was performed to evaluate the relationships between: CSF HIV-1 RNA viral load and CSF leukocyte count, protein concentration, CCPA titer, and HIV-1 plasma viral load; and between CSF leukocyte count and peripheral blood CD4+ T lymphocyte count. Data were processed with *Statistix *7.0 software (Analytical Software, Tallahassee, FL). Scatter plots were made using SPSS 15.0 software (Chicago, IL).

Paired, pre-treatment CSF and blood samples from 37 HIV-1 infected patients with culture-confirmed cryptococcal meningitis were collected. CSF samples from three patients were excluded due to the diagnosis of a simultaneous opportunistic CNS disease (either *Cytomegalovirus *encephalitis, cerebral toxoplasmosis, or Chagas' encephalitis). Therefore, the samples from 34 patients were used for the final analysis. The median (interquartile range) age was 35 years (32-42), and 74% of patients were male.

## Results

All data are presented as the median (interquartile range), unless otherwise specified. CD4+ T lymphocyte count was 24 cells/mm^3 ^(11-43). The CSF cytological and biochemical characteristics were as follows: leukocyte count, 10 cells/mm^3 ^(4-23); glucose, 39 mg/dL (32.7-50); protein concentration 0.75 g/L (0.48-1.06); and CCPA titer, 1/100 dilutions (1/10-1/1000). Eighty-five percent of patients had a positive CSF India ink examination.

HIV-1 plasma viral load was higher than CSF viral load, with values of 5.43 log_10 _copies/mL (4.96-5.87) and 4.83 log_10 _copies/mL (3.77-5.47), respectively (Wilcoxon signed-rank test, *p *= 0.001). There was no evidence of a statistical correlation between plasma and CSF viral load.

**Figure 1:**
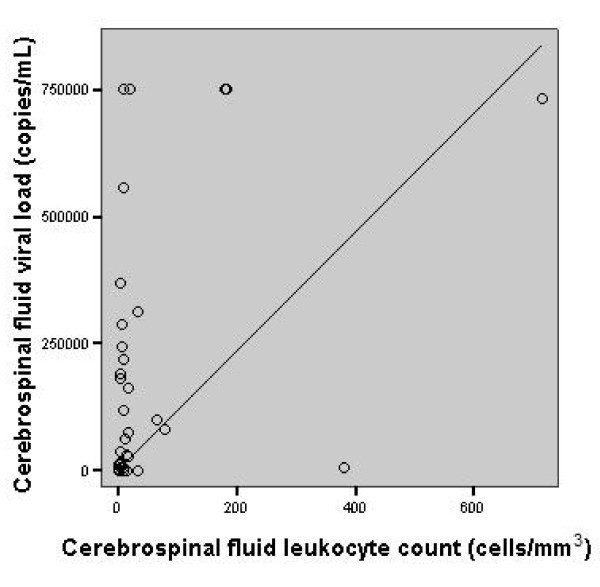
**Scatter plot graphic: correlation between cerebrospinal fluid leukocyte count and cerebrospinal fluid viral load (*r *= 0.4, *p *= 0.01) in patients with cryptococcal meningitis**.

There was a statistically significant correlation between CSF leukocyte count and CSF HIV-1 RNA viral load (*r *= 0.4, 95% CI = 0.13-0.63, *p *= 0.01), as shown in Figure 1. There was not a statistically significant correlation (*p *> 0.05) between CSF HIV-1 viral load and protein concentration, or CCPA titer (Table 1).

**Table 1:** Evaluation of variables that influence HIV-1 cerebrospinal fluid concentrations in cryptococcal meningitis: linear regression analysis

Variable	*r*	*p*	95% Confidence interval
HIV-1 plasma viral load	0.15	0.39	---------
**In CSF:**			
Leukocyte count	0.40	0.01	0.13 - 0.63
Protein concentration	0.28	0.09	---------
Cryptococcal antigen titer	-0.21	0.23	---------

**Figure 2:**
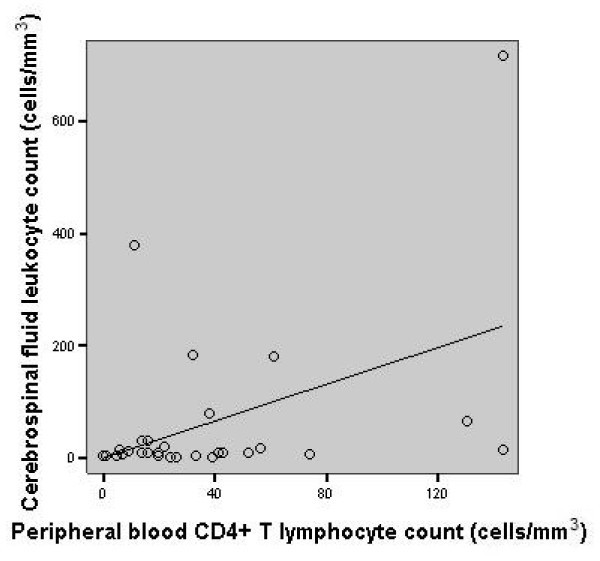
**Scatter plot graphic: correlation between peripheral blood CD4+ T lymphocyte count and the absolute CSF leukocyte count (*r *= 0.44; *p *= 0.0123) in patients with cryptococcal meningitis**.

Finally, a positive correlation was found between peripheral blood CD4+ T lymphocyte count and the absolute CSF leukocyte count (*r *= 0.44, 95% CI = 0.125-0.674, *p *= 0.0123), as shown in Figure 2.

## Discussion

Our study demonstrates that CSF leukocyte count is associated with CSF HIV-1 viral load in patients with meningitis caused by *Cryptococcus neoformans*. Although CSF leukocyte counts were low in our study population, there was a strong correlation between HIV-1 viral load and the number of leukocytes in the CSF. These findings suggest that, while CCPA antigen may inhibit the migration of leukocytes to the CNS [16], these inflammatory cells still contribute to viral load, and that the viral load of patients with this specific type of meningitis cannot be attributed to a spillover of virus from the plasma.

Cryptococcal meningitis is a chronic disease with an indolent course until the patient develops clinical symptoms. The chronicity of this process, with the development of a CSF inflammatory response, provides an environment for independent viral replication. The infiltrating leukocytes (which are predominantly lymphocytes) may harbour HIV-1 and thus constitute an exogenous source of the virus, contributing to the viral load at this site [13].

Our study also shows that viral load in cryptococcal meningitis is higher in plasma than in CSF, although no correlation was found between the plasma and CSF HIV-1 RNA viral loads. This is not unexpected, considering that such a correlation was described in patients with CD4 counts of >200 cells/mm^3 ^and the median CD4 T cell count of our population was 24 cells/mm^3^[7,12,21].

*In vitro *studies have shown that antigens from certain opportunistic organisms, such as CCPA from *Cryptococcus neoformans*, may promote viral replication [20]. This suggests that the presence of this organism (or its antigens) could directly enhance viral replication in addition to promoting an inflammatory response in the CNS. However, in the clinical setting of our investigation, no correlation was found between CCPA titer and CSF HIV-1 viral load in the linear regression analysis; that is, a higher antigen titer did not correlate with a higher viral load.

Therefore, our study tentatively suggests that the microorganism burden associated with a given CCPA titer may not be a determining factor of CSF HIV-1 viral load *in vivo*. To the best of our knowledge, this is the first study to assess the relationship between the levels of an opportunistic pathogen in CSF and HIV-1 viral load in a clinical setting.

A positive correlation was found between peripheral CD4+ T lymphocyte count and CSF leukocyte count. This finding is unexpected, considering the suppressed immune systems and modest CSF pleocytosis of our patient population. These results suggest that, despite the advanced level of immunodeficiency observed in patients with meningitis by *Cryptococcus neoformans*, peripheral blood CD4+ T lymphocyte counts influence the cellular response in the CSF.

Our study has several limitations. First, we used CCPA titer in the linear regression analysis to evaluate the correlation between the disease burden of this pathogen and CSF HIV-1 RNA viral load. Although previous reports demonstrated a strong correlation between cryptococcal colony-forming units in quantitative cultures and this antigen titer as measures of microorganism load in CSF [22], the first parameter would have been more accurate for our linear regression model.

Second, we did not measure cytokines in the CSF. Some molecules, such as TNF-α, IFN-γ, IL-6, and IL-8, are negatively correlated with baseline colony-forming units of *Cryptococcus neoformans *[23], but no studies to date have considered the potential influence these molecules may have on the CSF viral load in this disease. The study of cytokines in cryptococcal meningitis may further clarify the factors that determine CSF HIV-1 viral load in this context.

Third, our results are representative only of patients with meningitis caused by *Cryptococcus neoformans*, and cannot be extrapolated to other CNS infections, such as *Cytomegalovirus *encephalitis, tuberculous meningitis, progressive multifocal leukoencephalopathy, or cerebral toxoplasmosis.

Finally, the observational design of this study (chosen due to ethical constraints regarding the risks of the lumbar puncture procedure) precluded the inclusion of a control group of asymptomatic subjects (i.e., those without cryptococcal meningitis) with which to compare CSF viral loads.

## Conclusion

The present study shows that CSF HIV-1 viral load in patients with cryptococcal meningitis is positively correlated with CSF leukocyte count, and not with plasma viral load. The CSF cellular response may depend in part on the peripheral blood CD4+ T lymphocyte count, despite the advanced level of immunodeficiency observed in these patients, as a positive correlation was found between both variables. Further investigations are needed to elucidate the relationship between other CNS opportunistic infections and CSF HIV-1 viral load.

## Competing interests

The authors declare that they have no competing interests.

## Authors' contributions

DMC was responsible for the design of the study, patient enrolment, data analysis, and writing of the manuscript. AMC was responsible for the proceedings performed in the Virology Unit, and was co-writer of the manuscript. HR was responsible for patient enrolment. AA and RN were responsible for proceedings performed in the Mycology Unit. MBB undertook design of the study, and was supervisor of the proceedings performed in the Virology Unit, and co-writer and final supervisor of the manuscript. JAB undertook design and general supervision of the study, and was co-writer and final supervisor of the manuscript.

## References

[B1] Fierer DS Klotman ME Kidney and central nervous system as reservoirs of HIV infection Curr Opin HIV AIDS 2006 12 2 115 120 10.1097/01.COH.0000209581.88166.89 19372794

[B2] Pilcher CD Shugars DC Fiscus SA Miller WC Menezes P Giner J Dean B Robertson K Hart CE Lennox JL Eron JJ Jr Hicks CB HIV in body fluids during primary HIV infection: implications for pathogenesis, treatment and public health AIDS 2001 12 837 45 10.1097/00002030-200105040-00004 11399956

[B3] Bell JE An update on the neuropathology of HIV in the HAART era Histopathology 2004 12 549 59 10.1111/j.1365-2559.2004.02004.x 15569045

[B4] Spudich SS Nilsson AC Lollo ND Liegler TJ Petropoulos CJ Deeks SG Paxinos EE Price RW Cerebrospinal fluid HIV infection and pleocytosis: relation to systemic infection and antiretroviral treatment BMC Infectious Diseases 2005 12 98 10.1186/1471-2334-5-98 16266436 PMC1299327

[B5] Gisslén M Chiodi F Fuchs D Norkrans G Svennerholm B Wachter H Wachter H Hagberg L Markers of immune stimulation in the cerebrospinal fluid during HIV infection: a longitudinal study Scand J Infect Dis 1994 12 523 33 10.3109/00365549409011810 7855550

[B6] Price RW Staprans S Measuring the "viral load" in cerebrospinal fluid human immunodeficiency virus infection: window into brain infection? Ann Neurol 1997 12 675 78 10.1002/ana.410420502 9392565

[B7] Conrad AJ Schmid P Syndulko K Singer EJ Nagra RM Russell JJ Tourtellotte WW Quantifying HIV-1 RNA using polimerase chain reaction on cerebrospinal fluid and serum of seropositive individuals with and without neurologic abnormalities J Acquir Immune Defic Syndr Hum Retrovirol 1995 12 4 425 435 10.1097/00042560-199512000-00005 7583438

[B8] Christo PP Greco DB Aleixo AW Livramento JA HIV-1 RNA levels in cerebrospinal fluid and plasma and their correlation with opportunistic neurological diseases in a Brazilian AIDS reference hospital Arq Neuropsiquiatr 2005 12 907 13 16400403 10.1590/s0004-282x2005000600001

[B9] Gisslén M Fuchs D Svennerholm B Hagberg L Cerebrospinal fluid viral load, intrathecal immunoactivation, and cerebrospinal fluid monocytic cell count in HIV-1 infection J Acquir Immune Defic Syndr 1999 12 4 271 276 10428104 10.1097/00126334-199908010-00003

[B10] Hagberg L Forsman A Norkrans G Rybo E Svennerholm L Cytological and immunoglobulin findings in cerebrospinal fluid of symptomatic and asymptomatic human immunodeficiency virus (HIV) seropositive patients Infection 1988 12 13 18 10.1007/BF01646922 3360492

[B11] Yilmaz A Fuchs D Hagberg L Nillroth U Ståhle L Svensson JO Gisslén M Cerebrospinal fluid HIV-1 RNA, intrathecal immunoactivation, and drug concentrations after treatment with a combination of saquinavir, nelfinavir, and two nucleoside analogues: the M61022 study BMC Infectious Diseases 2006 12 63 10.1186/1471-2334-6-63 16566834 PMC1435910

[B12] Ellis RJ Gamst AC Capparelli E Spector SA Hsia K Wolfson T Abramson I Grant I McCutchan JA Cerebrospinal fluid HIV RNA originates from both local CNS and systemic sources Neurology 2000 12 927 36 10690988 10.1212/wnl.54.4.927

[B13] Morris L Silber E Sonnenberg P Eintracht S Nyoka S Lyons SF Saffer D Koornhof H Martin DJ High Human Immunodeficiency Virus Type 1 RNA Load in the Cerebrospinal Fluid from Patients with Lymphocytic Meningitis J Infect Dis 1998 12 473 76 10.1086/517379 9466541

[B14] Martin C Albert J Hansson P Pehrsson P Link H Sönnerborg A Cerebrospinal fluid mononuclear cell counts influence CSF HIV-1 RNA levels J Acquir Immune Defic Syndr Hum Retrovirol 1998 12 3 214 219 9495220 10.1097/00042560-199803010-00005

[B15] Brew BJ Pemberton L Cunningham P Law MG Levels of Human Immunodeficiency Virus type 1 RNA in Cerebrospinal Fluid Correlate with AIDS Dementia Stage J Infect Dis 1997 12 963 66 10.1086/514001 9086160

[B16] Buchanan KL Murphy JW What makes *Cryptococcus neoformans *a pathogen? Emerg Infect Dis 1998 12 71 83 10.3201/eid0401.980109 9452400 PMC2627665

[B17] Huffnagle GB McNeil LK Dissemination of *C. neoformans *to the central nervous system: role of chemokines, Th1 immunity and leukocyte recruitment J Neurovirol 1999 12 76 81 10.3109/13550289909029748 10190693

[B18] Chaka W Heyderman R Gangaidzo I Robertson V Mason P Verhoef J Verheul A Hoepelman AI Cytokine Profiles in Cerebrospinal Fluid of Human Immunodeficiency Virus-Infected Patients with Cryptococcal Meningitis: No Leukocytosis despite High Interleukin-8 Levels J Infect Dis 1997 12 1633 36 10.1086/517344 9395381

[B19] Garlipp CR Rossi CL Botín PV Cerebrospinal fluid in Acquired Immunodeficiency Syndrome with and without neurocryptococcosis Rev Inst Med Trop Sao Paulo 1997 12 6 323 325 9674282 10.1590/s0036-46651997000600003

[B20] Pettoello-Mantovani M Casadevall A Kollman TR Rubinstein A Goldstein H Enhancement of HIV-1 infection by the capsular polysaccharide of *Cryptococcus neoformans* Lancet 1992 12 21 23 10.1016/0140-6736(92)90142-p 1370335

[B21] Ellis RJ Hsia K Spector SA Nelson JA Heaton RK Wallace MR Abramson I Atkinson JH Grant I McCutchan JA Cerebrospinal fluid human immunodeficiency virus type 1 RNA levels are elevated in neurocognitively impaired individuals with acquired immunodeficiency syndrome Ann Neurol 1997 12 679 88 10.1002/ana.410420503 9392566

[B22] Brouwer AE Teparrukkul P Pinpraphaporn S Larsen RA Chierakul W Peacock S Day N White NJ Harrison TS Baseline correlation and comparative kinetics of cerebrospinal fluid colony-forming unit counts and antigen titers in cryptococcal meningitis J Infect Dis 2005 12 681 84 10.1086/432073 16028138

[B23] Siddiqui AA Brouwer AE Wuthiekanun V Jaffar S Shattock R Irving D Sheldon J Chierakul W Peacock S Day N White NJ Harrison TS IFN gamma at the site of infection determines the rate of clearance of infection in cryptococcal meningitis J Immunol 2005 12 1746 50 15661940 10.4049/jimmunol.174.3.1746

